# Efficiency of a Seedling Phenotyping Strategy to Support European Wheat Breeding Focusing on Leaf Rust Resistance

**DOI:** 10.3390/biology10070628

**Published:** 2021-07-06

**Authors:** Ulrike Beukert, Nina Pfeiffer, Erhard Ebmeyer, Valentin Hinterberger, Stefanie Lueck, Albrecht Serfling, Frank Ordon, Albert Wilhelm Schulthess, Jochen Christoph Reif

**Affiliations:** 1 Institute for Resistance Research and Stress Tolerance, Julius Kuehn-Institute (JKI), 06484 Quedlinburg, Germany; ulrike.beukert@julius-kuehn.de (U.B.); albrecht.serfling@julius-kuehn.de (A.S.); frank.ordon@julius-kuehn.de (F.O.); 2 KWS LOCHOW GmbH, 29303 Bergen, Germany; nina.pfeiffer@kws.com (N.P.); erhard.ebmeyer@kws.com (E.E.); 3 Department of Breeding Research, Leibniz Institute of Plant Genetics and Crop Plant Research (IPK), 06466 Gatersleben, Germany; hinterberger@ipk-gatersleben.de (V.H.); lueck@ipk-gatersleben.de (S.L.); schulthess@ipk-gatersleben.de (A.W.S.)

**Keywords:** wheat leaf rust, phenotyping, seedling resistance, adult plant resistance, detached leaf assay

## Abstract

**Simple Summary:**

Leaf rust resistance is of high importance for the European wheat production in order to avoid yield and quality losses. Modern breeding is aiming to maximize the selection gain within a short time period. To realize a successful resistance breeding, detailed knowledge about the genetic architecture of leaf rust resistance, as well as a precise and fast phenotyping strategy, are necessary. The examination of detached leaf assays of juvenile plants inoculated under controlled conditions and phenotyped by a robotic-based, high-throughput system is a promising approach in this respect. Known leaf rust resistance genes showing qualitative or quantitative effects and their expression starts at different developmental stages of wheat. Therefore, this study validated the transferability of results from detached leaf assays to the field and assessed the benefits of this phenotyping strategy to support leaf rust resistance breeding. Phenotyping detached leaves of wheat seedlings by using an automated, high-throughput methodology is a valuable tool to improve leaf rust resistance.

**Abstract:**

Leaf rust resistance is of high importance for a sustainable European wheat production. The expression of known resistance genes starts at different developmental stages of wheat. Breeding for resistance can be supported by a fast, precise, and resource-saving phenotyping. The examination of detached leaf assays of juvenile plants inoculated under controlled conditions and phenotyped by a robotic- and computer-based, high-throughput system is a promising approach in this respect. Within this study, the validation of the phenotyping workflow was conducted based on a winter wheat set derived from Central Europe and examined at different plant developmental stages. Moderate Pearson correlations of 0.38–0.45 comparing leaf rust resistance of juvenile and adult plants were calculated and may be mainly due to different environmental conditions. Specially, the infection under controlled conditions was limited by the application of a single rust race at only one time point. Our results suggest that the diversification with respect to the applied rust race spectrum is promising to increase the consistency of detached leaf assays and the transferability of its results to the field.

## 1. Introduction

Wheat leaf rust, caused by *Puccinia triticina*, is a very important fungal disease [1] because its epidemics are able to affect both grain yield and grain quality [2]. Such crop losses can be avoided by breeding and growing resistant wheat cultivars. Approximately 90 genes have been identified as the cause of leaf rust resistance [3], with *Lr1*, *Lr3a*, *Lr10*, *Lr13*, *Lr14a*, *Lr17b*, *Lr20*, *Lr26*, and *Lr37* in particular being intensively used in European varieties [4,5,6]. The majority of known leaf rust resistance genes cause seedling resistance (also called race-specific or qualitative resistance) [7,8], which is typically expressed throughout the plant life cycle and results in a hypersensitive response or programmed cell death [7]. The resistance genes *Lr1*, *Lr3*, *Lr10*, *Lr11*, *Lr14a*, and *Lr24* are present with high frequency within the European wheat germplasm [9], but are typically effective against only a limited number of leaf rust races. Due to its monogenic inheritance, seedling resistance follows Flor’s gene for gene concept [10,11] and can be easily broken by the permanent evolution of new leaf rust virulences [7,12,13]. In contrast, *Lr34* and *Lr46* are known to cause quantitative resistance of high durability [14,15], while approximately 20 different quantitative trait loci (QTL) were identified, resulting in partial resistance [16]. Quantitative resistance is non-race-specific and based on a few minor genes effecting each a reduction of infestation, while combining some minor genes within the same variety resulted in a stable and long-lasting efficiency of leaf rust resistance [7]. Due to its late expression after the seedling stage of plants, this resistance mainly leads to adult plant resistance (APR), which rarely can be also caused by qualitative resistance genes [17,18]. The full expression of adult plant resistance (APR) efficiency is highly variable within the plant life cycle and takes place between tillering and heading (EC20-EC59). This behavior can be influenced by the factors environment, temperature, genetic background, intensity, and aggressiveness of occurring leaf rust races [18,19].

The expected dynamic of expanding diseases and their increased importance for the European wheat growing area resulted in rising requests to resistance breeding. The main goal of breeding is to maximize selection gain per unit of time, which would shorten breeding cycles and thus, allow a faster adaption of grown varieties to altering conditions on farmers’ fields [20]. During the last decades, implementing modern molecular marker techniques supported a rapid and efficient wheat breeding. Nowadays, this development can be intensified by the integration of promising tools, such as speed-breeding protocols or genome-wide prediction strategies within the breeding process [20,21,22]. While a successful breeding strategy is based on highly precise phenotypic data, it is necessary, but challenging, to generate robust information within the dramatically reduced breeding cycle [20]. Therefore, the development of pioneering phenotyping strategies is requested to generate highly precise data in a time- and resource-saving manner [20]. Some strategies fulfilling these issues have already been published on the basis of greenhouse trials [23,24]. For instance, Lueck et al. (2020) [23] reported a highly promising approach by generating detached leaf assays of seedlings screened by a robotic- and computer-based, high-throughput system (further denoted as Macrobot platform). This methodology allows a fast resistance quantification for a large number of single plants using steadily updated pathogen populations or observing in parallel different diseases or isolates within separated experiments. To verify the benefit of this modern phenotyping procedure to practical wheat breeding aiming at leaf rust resistance, validation is needed examining the transferability of results generated by observing detached leaves of juvenile greenhouse plants to adult plants grown under field conditions [25].

The aims of this study were to (1) investigate the resistance of European wheat elite lines at different juvenile plant stages against an aggressive leaf rust race compared to adult plants tested in field trials with naturally occurring leaf rust infections, (2) infer the genetic architecture of leaf rust resistance based on the detected correlations between seedling and adult plant resistance, and to (3) validate the workflow including greenhouse seedling tests combined with a high-throughput phenotyping platform to support resistance breeding mainly focusing on adult plant resistance against leaf rust.

## 2. Materials and Methods

### 2.1. Plant Material

The study is based on 240 European elite winter wheat (*Triticum aestivum* L.) lines of the breeding program of KWS LOCHOW GmbH (Bergen, Germany). The 240 genotypes were selected on the basis of preliminary disease resistance data and reflect a broad phenotypic diversity with respect to resistance against leaf rust. No further information on the presence of particular resistance genes in the 240 genotypes was available.

### 2.2. Evaluating Leaf Rust Resistance of Adult Plants in Field Trials

The examined 240 genotypes were evaluated together with 1586 additional wheat lines for naturally occurring leaf rust resistance. The data is not orthogonal and includes field trials conducted in 5 and 4 environments in the years 2017 and 2018, respectively (for details, see Table 1). Five released varieties (*Bonanza*, *Elixer*, *Julius*, *Nordkap*, *RGT Reform*) were included as checks in all field trials enabling joint analyses. Plot size ranged from 0.5 m^2^ to 17.25 m^2^, while different experimental designs were used, i.e., alpha-lattice, randomized complete blocks, or adjustment through a moving grid to correct for uncontrolled spatial variation. Infection of genotypes with leaf rust occurred naturally and was scored at the date of flowering (EC 65) on the flag leaf. An ordinal scale from 1 to 9, according the Bundessortenamt (2000) [26], was used in order to score infections, where 1 stands for minimal symptoms and 9 indicates extensive disease symptoms.

### 2.3. Evaluating Leaf Rust Resistance in Greenhouse Experiments

The 240 genotypes were additionally phenotyped in replicated greenhouse experiments using detached leaf assays of 10-day old seedlings (EC 12; T1), for which 7 leaves per genotype were observed. The susceptible standard variety *Borenos* was used as control. Inoculation was implemented in an air-blowing inoculation tower using uredospores of the aggressive leaf rust isolate *77 WxR* [27]. After an incubation of 8 days under controlled temperature conditions (16 °C), visibly differentiable leaf rust symptoms appeared on the leaf segments. Standardized imaging of infected leaf assays was then realized by the automated phenotyping platform Macrobot, while the BluVision software was used to analyze the images. Briefly, the software detects leaf samples and uredospore pustules based on the color and measures their pixel sizes. The relation between leaf size and pustule size resulted in the rated percentage of infected leaf area [28]. A more detailed description of the experimental setup can be found elsewhere [23].

We examined the influence of the developmental stage on the leaf rust resistance scoring focusing on 40 genotypes. They were selected in order to portray the variation observed for the 240 genotypes. The 40 genotypes were grown under greenhouse conditions for 5 (EC 19; T2), and 10 weeks (EC 25; T3), each in two replications. Subsequently, resistance screening was undertaken using detached leaf assays and the above described procedure.

### 2.4. Analyses of Data from Field Trials

Phenotypic data were analyzed using a two-step approach [29]. Each trial times location combination was used to perform a quality check implementing the following linear mixed model:

(1) $$y_{def}=\mu +g_{d}+r_{e}+b_{f\left(e\right)}+\varepsilon_{def},$$

where $y_{def}$ represents the performance of the $d^{th}$ genotype at the $f^{th}$ block within the $e^{th}$ replication, μ is the intercept, $g_{d}$ stands for the genotype effect, $r_{e}$ represents the replication, $b_{f\left(e\right)}$ symbolizes the effect of the $f^{th}$ block nested within the $e^{th}$ replication, and $\varepsilon_{def}$ is the corresponding residual. The calculation of repeatability was completed for replicated trials based on the estimated variance components as:

(2) $$r=\frac{\sigma_{Genotype}^{2}}{\sigma_{Genotype}^{2}+\frac{\sigma_{error}^{2}}{No.\;of\;replicates}}.$$

Trial by location combinations with a repeatability >0.4 were taken to obtain the best linear unbiased estimations (BLUEs) applying the following linear mixed model:

(3) $$y_{dlef}=\mu +g_{d}+t_{l}+r_{e\left(l\right)}+b_{f\left(e\right)}+\varepsilon_{dlef},$$

where $y_{dlef}$ stands for the performance of the $d^{th}$ genotype at the $l^{th}$ trial in the $f^{th}$ block of the $e^{th}$ replication, μ is the intercept, $g_{d}$ is the genotype effect, $t_{l}$ represents the trial, $r_{e\left(l\right)}$ symbolizes the replication nested within the $l^{th}$ trial, $b_{f\left(e\right)}$ represents the block effect nested within the $e^{th}$ replication and the $l^{th}$ trial, while $\varepsilon_{dlef}$ is the corresponding residual. The trial effect was excluded for locations with only one trial.

The BLUEs of genotypes for every single location were combined and the following linear mixed model was fitted:

(4) $$y_{dm}=\mu +g_{d}+l_{m}+\varepsilon_{dm},$$

where $y_{dm}$ symbolizes the phenotypic observation of the $d^{th}$ genotype in the $m^{th}$ location, μ is the intercept, $g_{d}$ represents the genotype effect, while $l_{m}$ stands for the location effect and $\varepsilon_{dm}$ is the corresponding residual. Heritability was calculated as the ratio between the variance of the genotype versus the phenotype following standard procedures [30]. In addition, the correlation between single field environments and the serial mean except the examined environment was analyzed.

### 2.5. Analyses of Data from Greenhouse Experiments

A statistical workflow developed by Hinterberger et al. (2021) [31] was implemented to analyze infestation data of single leaf segments in order to identify unreliable data points and inoculation groups. Curated data were used to estimate variance components based on the following linear mixed model:

(5) $$y_{dno\;}=\mu +a_{n}+g_{d}+G_{d}\;x\;a_{n}+I_{o}+G_{d\left(o\right)}+\varepsilon_{dop},$$

where $y_{dno\;}$ is the average infected leaf area of the $d^{th}$ genotype at the $n^{th}$ developmental stage and tested within the $o^{th}$ inoculation group, μ denotes the common mean, $a_{n}$ symbolizes the $n^{th}$ level of plant developmental stage, $g_{d}$ indicates the main effect of the $d^{th}$ genotype, $G_{d}\;x\;a_{n}$ is the interaction of the $d^{th}$ genotype with the $n^{th}$ plant stage, $I_{o}$ accounts for the effect of the $o^{th}$ inoculation group, $G_{d\left(o\right)}$ is the $d^{th}$ genotype nested within the $o^{th}$ inoculation group, and $\varepsilon_{dop}$ denotes the error term of the model. For variance components estimation, all effects in Equation (5), excepting μ  and $a_{n}$, were treated as random. Firstly, the calculation of heritability examining the whole data set was realized based on the following equation:

(6) $$h^{2}=\frac{\sigma_{Genotype}^{2}}{\left(\sigma_{Genotype}^{2}+\frac{\sigma_{Genotype\;x\;Age}^{2}}{No.\;of\;age\;levels}+\frac{\sigma_{error}^{2}}{No.\;of\;replicates}\right)}.$$

Later, the estimation of BLUEs and belonging standard errors was realized separately for each plant level as

(7) $$y_{dno\;}=\mu +g_{d}+I_{o}+G_{d\left(o\right)}+\varepsilon_{dop},$$

treating μ and $g_{d}$ as fixed effects, while the remaining factors were considered as random. The heritability was calculated separately for each developmental stage as:

(8) $$h^{2}=\frac{\sigma_{Genotype}^{2}}{\sigma_{Phenotype}^{2}}=\frac{\sigma_{Genotype}^{2}}{\left(\sigma_{Genotype}^{2}+\frac{\sigma_{error}^{2}}{No.\;of\;replicates}\right)}.$$

The estimated BLUEs out of greenhouse experiments were contrasted with the BLUEs of field phenotyping by calculating the Pearson correlation coefficients. Every step of statistical analysis was performed using R software [32] in combination with the package ASReml-R 3.0 [33].

## 3. Results

### 3.1. Extensive Field Trials Resulted in Precise Estimates of Adult Plant Resistance against Leaf Rust

The 240 wheat lines were evaluated for leaf rust resistance in 5 and 4 German environments within the years 2017 and 2018, respectively. The mean infestation class of controls ranged between 3 and 6 in dependence of the special variety, while the application of statistical models adjusted the results of varying field conditions. The data quality of replicated trial times location combinations was examined by estimating the repeatability for the individual environments. Repeatability of trials conducted in 2017 ranged from 0.45 to 0.93, while analysis of the 2018 field trials resulted in repeatabilites between 0.57 and 0.96 (Table 2). The correlation between overlapping genotypes of the individual environments averaged r  = 0.40 (Table 3). The combined analyses of field trials resulted in a broad-sense heritability of $h^{2}$ = 0.9. Leaf rust resistance of germplasm tested under field conditions spanned a range of 7.67 with a mean value of 3.43 (Table S1).

### 3.2. Ensuring Stable Pathogen Pressure in Greenhouse Experiments Is Challenging

The analysis of the raw data highlighted the challenge to guarantee a stable disease pressure under greenhouse conditions. We therefore had to exclude 50% of the inoculation groups, because the mean of the susceptible controls per inoculation group was below 2% for the infected leaf area. The most challenging factor was to generate leaf rust spores of consistent aggressiveness under variable greenhouse conditions, due to the climatic variation of the seasons (Figure S1). Moreover, infection and the development of uredospore pustules were highly dependent on light and temperature conditions during inoculation and incubation. After quality control, data for 240, 40, and 41 genotypes remained, belonging to the different plant stage clusters of T1, T2, and T3, respectively. A large proportion of the phenotypic variance was explained by genotype (22%) and interaction effects between genotypes and plant developmental stages (24%) in the integrated analysis across all plant stages (Table 4). The broad-sense heritability amounted to $h^{2}$ = 0.64. The estimated leaf rust resistance ranged from 0.3% to 26.7% of infected leaf area with a mean of 1.4%.

**Figure 1:**
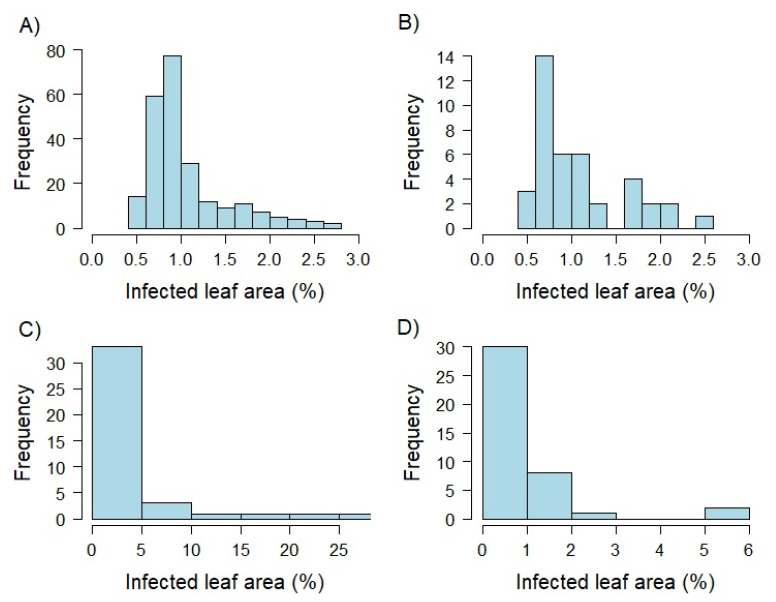
Histograms showing the distribution of best linear unbiased estimates (BLUEs) based on detached leaf assays produced within greenhouse experiments. Percentage of infected leaf area was determined by an automated phenotyping platform using image analysis. The whole genotype set was examined as two leaf seedlings (EC 12; **A**). In addition, a limited set of 40 genotypes was tested 10 days (EC 12; **B**), 5 weeks (EC 19; **C**), and 10 weeks (EC 25; **D**) after sowing.

Analyzing variance components separately for each developmental stage set based on the selected 40 genotypes resulted in high amounts of phenotypic variance explained by the interaction of inoculation group with the genotype (14–47%), and the inoculation group (9–28%) for plant stage sets T1 and T3. In contrast to that, the factors genotype (83%) and residual (46%) accounted for the highest proportion of total variance for the T2 and T3 sets, respectively. Broad-sense heritability ranged from $h^{2}$ = 0.58 to 0.92 for the three different plant developmental stages. The highest $h^{2}$ value was realized at 0.92 at the developmental stage T2, while T1 and T3 revealed similar values of $h^{2}$ = 0.58 to 0.60. Plants of the developmental stage T2 showed the widest range of infected leaf area resulting in the highest mean value compared to the remaining groups (Figure 1, Table S1). Comparing leaf infection of T1 and T3 lead to similar ranges and means.

### 3.3. Seedling Resistance Showed a Significant Correlation to Adult Plant Resistance

**Figure 2:**
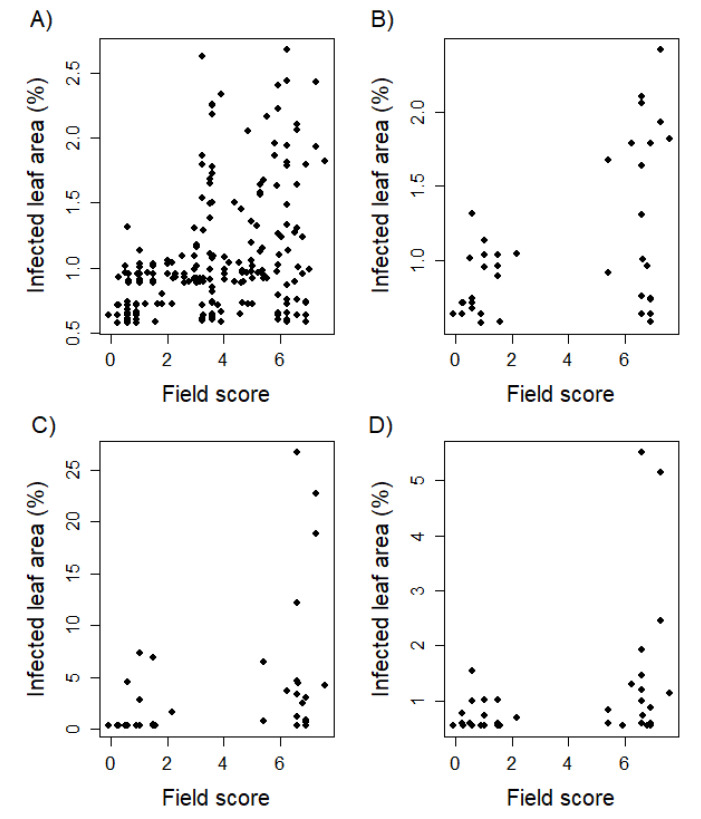
Biplots comparing leaf rust resistance of juvenile plants with adult plants phenotyped in the field environment. Phenotyping in the field was realized following an ordinal scale of increasing infestation from 1 to 9. Phenotypic data was statistically adjusted applying a linear mixed model resulting in a broaden score range. The whole genotype set was examined within greenhouse trials as two leaf seedlings (EC 12; **A**). In addition, a limited set of 40 genotypes was tested 10 days (EC 12; **B**), 5 weeks (EC 19; **C**), and 10 weeks (EC 25; **D**) after sowing.

Correlating seedling resistance evaluated in greenhouse experiments with adult plant resistance rated in field trials resulted in values ranging from r = 0.34 to 0.48 (Table 5). Leaf rust infection of the developmental stage T1 showed the highest Pearson correlation to field phenotyping amounting to r = 0.48 (*p* < 0.01). The Pearson correlation between greenhouse and field data decreased with increasing developmental stages of the tested greenhouse plants. There is a noticeable cluster of 8 genotypes showing susceptibility as adult plants on the field, but resistance as seedlings within greenhouse trials (Figure 2). Analyzing the correlation of resistance scores based on single field environments and the serial mean, except the examined environment, resulted in a range from r = 0.13 to 0.74 with an average Pearson correlation of r = 0.57 (Table 6).

## 4. Discussion

### 4.1. Divergent Conditions Increase the Quality of Resistance Phenotyping within Controlled Environments

The same inoculation material was used for all greenhouse experiments; however, there were variable environmental conditions in the standard greenhouse used. The latter was confirmed by a genotype x inoculation group variance component explaining 14.2–46.5% of the total variance (Table 4). This demonstrates the importance of replicated data to ensure high precision of phenotyping in greenhouse trials. However, high precision is not sufficient and screening with the detached leaf assay should allow to mimic the field situation to generate relevant information for applied resistance breeding [25]. Our study revealed moderate correlations between seedling and adult plant resistance ranging from r = 0.34 to 0.48 (Table 5). Previous studies reported lower values of 0.17–0.29 [34] and 0.24–0.27 [35] for leaf rust resistance observed in an American spring wheat and a multi-parental winter wheat population based on European elite cultivars, respectively. Thus, our results suggest a closer relationship between seedling and adult plant resistance than reported earlier; however, values are still low when relying solely on detached leaf assays for selection.

**Figure 3:**
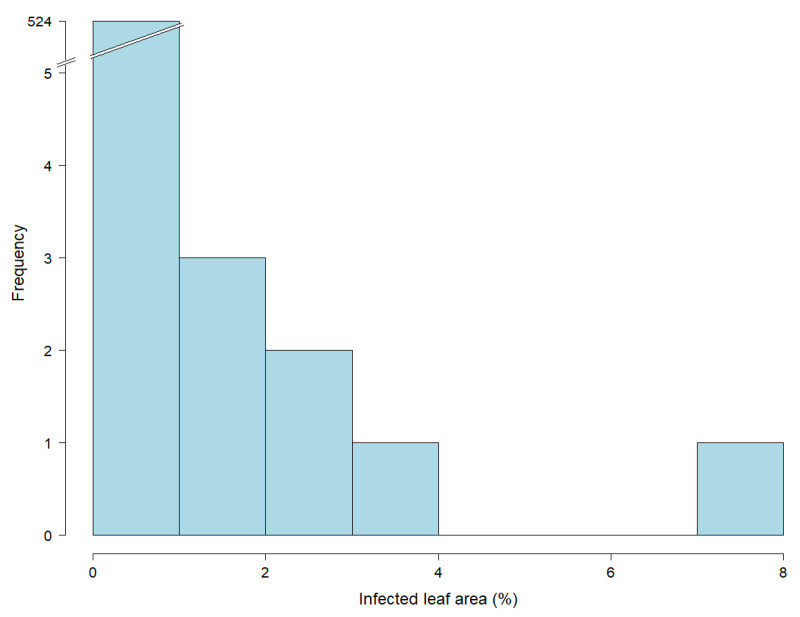
Histogram showing the distribution of leaf rust infections based on 532 leaf samples of 15-week old greenhouse plants (EC 32). Percentage of infected leaf area was determined by an automated phenotyping platform using image analysis. Due to the low infection rates (see the truncated bar of the histogram), data of this developmental stage were excluded from statistical analysis.

The key challenge is therefore to further increase the correlation between greenhouse and field trials, which is often associated with seedling versus adult plant resistance [36]. One approach may be to test plants of a later developmental stage in greenhouse trials, which was conducted in this study, but did not increase the correlation when the greenhouse trials were conducted for 5- (EC 19) or 10-week old plants (EC 25) (Figure 3). We also attempted to go beyond this time point; however, leaf senescence on old leaves prevented quantification of rust infection. Interestingly, the highest correlation was observed at the youngest plant developmental stage (Table 5), i.e., 10-day old seedlings (EC 12), which can be attributed to an adequate range of infected leaf area (Table 4). This can be explained by the fact that young leaves with low differentiation are able to cope with a change of circumstances and adapt better to the conditions of detached leaf assays [37]. Leaf senescence has been confirmed as a challenging factor in previous studies with detached leaf assays [38,39]. Senescence occurred primarily on aged leaves as a result of stress and is associated with the presence of phytohormones [39]. Meaningful leaf rust phenotyping examining plants at later developmental stages (>5 weeks) in greenhouse experiments is very difficult, and implemented trials did not yield evaluable results or methodical advances [40].

Another approach to increase the correlation between greenhouse and field trials could be to increase the diversity of used rust isolates [34]. Although the highly aggressive leaf rust isolate *77 WxR* was applied, which exhibits virulence in juvenile plant stages against several resistance genes, viz. *Lr1*, *Lr2a*, *Lr2b*, *Lr2c*, *Lr3a*, *Lr3bg*, *Lr3ka*, *Lr4*, *Lr11*, *Lr12*, *Lr13*, *Lr14a*, *Lr14b*, *Lr15*, *Lr17*, *Lr17b*, *Lr18*, *Lr20*, *Lr22a*, *Lr22b*, *Lr23*, *Lr26*, *Lr33*, *Lr35*, *Lr36*, *Lr37*, *Lr38*, and *Lr49* [27], natural selection pressure leads to divergent isolate populations resulting in differences between greenhouse and field trials. However, there are also differences between field trials. Looking at the relevant comparisons, i.e., correlation at the single location versus the series except the location under consideration, there is an average correlation of r = 0.57 (Table 6). This is only 16% lower compared to the correlation between greenhouse and field trials. This suggests that an increase in correlation between field and greenhouse trials is possible by using different rust isolates in the latter.

To further investigate the possibilities and limitations to increase the correlation between greenhouse and field environments, we looked at the outliers: genotypes that were susceptible as juvenile plants but resistant at the adult plant stage were absent in this study. Nevertheless, 8 lines that were resistant at a juvenile stage were susceptible under field conditions (Figure 2). The pedigrees of the genotypes are known and include susceptible parents for inconsistent lines (data not shown). Moreover, 5 of those lines show an early ripening (data not shown), avoiding the confrontation with a highly increased leaf rust pressure after many cycles of uredospore multiplication [41]. Therefore, the moderate correlation between seedling and adult plant resistance was supported by contrasting virulence differences of a diverse pathogen population in the field, compared to the limited range of a single isolate used for artificial inoculation. This again supports the hypothesis that the use of mixtures of relevant virulent isolates can help to approximate greenhouse tests of seedlings to field trials. To conduct greenhouse screening with more diverse rust isolates, more information is needed on the composition of naturally occurring rust populations for different wheat growing areas and the conditions to simulate these rust populations in greenhouse trials.

### 4.2. Examining Seedling Resistance Could Support Leaf Rust Resistance Breeding within European Wheat

Test conditions within field trials are similar to common agricultural growing environments and phenotyping is able to detect relevant resistances comprising seedling, as well as adult plant resistance, while they cannot be clearly distinguished under field conditions. Studies reporting an increased effectiveness at the adult plant stage were missing for the most common resistance genes of European wheat cultivars. Boyd et al. (2006) [42] detected adult plant leaf rust resistance, which was absent at the juvenile stage, by examining mutant lines based on the wheat cultivar *Hobbit ‘sib’*. Such a behavior may be explained by the late expression of adult plant resistance taking place after testing [24,42]. The regulation of resistance expression in dependence of the plant developmental stage is well known in wheat [43] and was also reported for its rust resistance [24,42].

In contrast to field experiments, it is more difficult to detect adult plant resistance by performing greenhouse trials examining juvenile plants, while seedling resistance could be discovered easily at early plant developmental stages. It is known that the majority of discovered leaf rust resistance genes cause seedling resistance, while *Lr1*, *Lr3*, *Lr10*, *Lr11*, *Lr12*, *Lr13*, *Lr14a*, and *Lr24* are frequently used within the European wheat germplasm [9]. The importance of detecting seedling resistance was underlined by their reduced, but continuous participation on leaf rust resistance, even after their break down [27]. Therefore, greenhouse observations of juvenile plants could support breeding for leaf rust resistance in European wheat.

The simultaneous examination of leaf rust resistance within greenhouse and field trials helps to distinguish between seedling and adult plant resistance. A deeper examination of inconsistent genotypes would be of high relevance understanding the genetic architecture of leaf rust resistance and support resistance breeding in the examined wheat population. Breeders are confronted with the strong effect, as well as the simple handling, of qualitative resistance genes combined with a short durability, which is contrary for quantitative resistances and can be primarily summarized as seedling versus adult plant resistance, respectively [11,27].

If an increased correlation between greenhouse and field evaluation can be accomplished in the future, the greenhouse workflow affords promising advantages for the application within the breeding process. Experiments with juvenile plants would be beneficial to generate phenotypic data of a large genotype set with a highly desired advantage in time. The appearance of intended inoculum can be ensured, while the occurrence of other pathogens causing masking effects on the trait of interest can be excluded.

### 4.3. Automated Phenotyping of Detached Juvenile Leaves Is Beneficial for Resistance Breeding

Speed-breeding is an important method to improve cereal breeding, and protocols were already established for spring wheat [20,44]. Due to a plant growing under controlled conditions and a highly elongated photoperiod, growing time per generation was extremely shortened [44]. Its combination with modern genomic-based selection methods opens new opportunities for resistance breeding, e.g., fast trait introgression combining high yield and multiple resistance or stacking of resistance genes [20].

Due to their high durability, quantitative leaf rust resistance genes are highly desired [7]. However, they are mostly expressed in adult plants [17,18], resulting in a time-consuming identification with missed distinction to seedling resistance. Therefore, the identification and stacking of seedling resistance could be an efficient alternative.

In addition, fast and efficient phenotyping systems are required to support the application of speed-breeding [20,24]. Within this study, a phenotyping workflow examining detached leaves of greenhouse seedlings was applied by an automated, high-throughput platform. This system is time- and resource-saving, generating precise quantitative data of leaf rust resistance.

The used phenotyping workflow was conceived aiming at an optimal examination of detached seedling leaves. The growing time of plants in addition to 8 days of rust incubation is needed to finally quantify the leaf rust symptoms, while screening results can be checked by facing the automatically saved images. Most of the resistance mechanisms of adult plants are still present in seedlings [9], and resistance can be identified based on an image analysis which is sensible even for low infection levels.

The implemented phenotyping strategy is relevant to support resistance breeding, especially when it is combined with a speed-breeding protocol. As an outlook, the applied greenhouse workflow provides some further interesting opportunities improving resistance screening. Experiments can be performed over a wide range of the year. A simultaneous examination of different leaf diseases or pathogen isolates can be realized within separated experiments. The image analysis software is available for quantifying leaf rust, stripe rust and powdery mildew [28].

## Figures and Tables

**Table 1:** Characterization of field environments, in which leaf rust resistance was evaluated.

	Nasenberg	Nörvenich	Seligenstadt1	Seligenstadt	Söhnke- Nissenkoog	Wetze1	Wetze2	Wetze	Wohlde
Elevation above sea level (m)	148	106	283	283	1	136	136	136	73
Average annual temperature (°C)	10.69	11.25	11.02	11.02	9.77	10.13	10.13	10.13	9.94
Average annual precipitation (mm)	548.54	548.66	551.36	551.36	902.50	584.92	584.92	584.92	740.14
Year (s)	2017, 2018	2018	2017, 2018	2017	2017	2017, 2018	2018	2017, 2018	2017
Plot size (m^2^)	17.25	7.4	0.5	14.85, 6.6	0.5	0.5	0.5	14.25, 6.25	0.5
Field design	Alpha-lattice	Alpha-lattice	Randomized complete block	Alpha-lattice, Moving grids	Randomized complete block	Randomized complete block	Randomized complete block	Alpha-lattice, Moving grids	Randomized complete block
No. of genotypes	116, 119	119	780, 1138	64	56	780, 1135	1138	93, 64	780
No. of trials	2	2	10	1	1	10, 11	11	2, 11	10
Replications	1	1	2	1	2	2, 1	2	1, 2	2
Genotypes per trial	64	64	42–140	64	56	42–140	64–140	64, 64–140	42–140
Genotypes of selected set	27, 26	26	126, 128	19	5	126, 127	128	14, 17	126

**Table 2:** Repeatability of replicated field trials. Trials with a repeatability below 0.4 were excluded before performing further analyses.

	Seligenstadt1 2017	Söhnke-Nissenkoog 2017	Wetze1 2017	Wohlde 2017	Seligenstadt1 2018	Wetze2 2018
Trial1	0.82	-	0.70	0.59	0.93	0.76
Trial2	0.88	-	0.75	0.54	0.89	0.77
Trial3	0.93	0.56	0.51	0.63	-	-
Trial4	0.89	-	0.71	0.14	0.94	0.77
Trial5	0.89	-	0.45	0.50	0.96	0.82
Trial6	0.92	-	0.48	0.62	-	-
Trial7	0.77	-	0.59	0.25	0.91	0.76
Trial8	0.67	-	0.53	0.12	0.93	0.65
Trial9	0.79	-	0.60	0.22	0.91	0.78
Trial10	0.91	-	0.60	0.51	-	0.59
Trial11	-	-	-	-	0.90	0.57
Trial12	-	-	-	-	0.84	0.68
Trial13	-	-	-	-	0.93	0.76

**Table 3:** Pearson correlation of leaf rust scores observing overlapping genotypes of pairwise compared field environments, while the number of overlapping genotypes is given in brackets.

	Seligenstadt1 2017	Seligenstadt 2017	Söhnke- Nissenkoog 2017	Wetze1 2017	Wetze 2017	Wohlde 2017	Nasenberg 2018	Nörvenich 2018	Seligenstadt1 2018	Wetze1 2018	Wetze2 2018	Wetze 2018
Nasenberg 2017	0.60 *** (116)	0.56 *** (64)	−0.30 (17)	0.56 *** (116)	0.46 *** (93)	0.42 *** (116)	0.14 (44)	0.06 (44)	0.39 ** (46)	0.26 (46)	0.14 (46)	0.61 * (12)
Seligenstadt1 2017	-	0.65 *** (64)	0.28 * (56)	0.64 *** (780)	0.64 *** (93)	0.53 *** (352)	0.45 *** (84)	0.34 ** (84)	0.69 *** (92)	0.53 *** (92)	0.56 *** (92)	0.48 *** (46)
Seligenstadt 2017		-	−0.31 (9)	0.63 *** (64)	0.46 *** (51)	0.48 *** (64)	0.21 (16)	0.72 ** (16)	0.31 (17)	0.50 * (17)	0.41 (17)	0.70* (11)
Söhnke- Nissenkoog 2017			-	0.21 (56)	0.39 (15)	0.18 (56)	−0.04 (17)	0.20 (17)	0.19 (21)	0.43 (21)	0.29 (21)	0 (8)
Wetze1 2017				-	0.54 *** (93)	0.53 *** (352)	0.48 *** (84)	0.04 (84)	0.73 *** (92)	0.48 *** (92)	0.53 *** (92)	0.47 *** (46)
Wetze 2017					-	0.40 *** (93)	0.33 * (37)	−0.12 (37)	0.74 *** (39)	0.68 *** (39)	0.57 *** (39)	0.58 (10)
Wohlde 2017						-	0.53 *** (59)	0.17 (59)	0.40 ** (65)	0.51 *** (65)	0.33 ** (65)	0.41 (21)
Nasenberg 2018							-	0.14 (119)	0.67 *** (119)	0.52 *** (119)	0.54 *** (119)	0.50 *** (64)
Nörvenich 2018								-	0.04 (119)	0.16 (119)	0.06 (119)	0.43 *** (64)
Seligenstadt1 2018									-	0.64 *** (1005)	0.73 *** (1008)	0.52 *** (64)
Wetze1 2018										-	0.59 *** (1135)	0.47 *** (64)
Wetze2 2018											-	0.39 *** (64)

Significant at * *p*-value ≤ 0.05, ** ≤ 0.01, *** ≤ 0.001. Otherwise, not significant.

**Table 4:** Variance components analysis, estimation of heritability (*h^2^*), and distribution of analyzed infected leaf area based on greenhouse data. Analysis was performed observing different scenarios, examining the whole data set including information of all plant developmental stages (Analysis with whole data set) in contrast to single analysis of each stage. The full genotype set (n= 240) at plant stage T1 (All genotypes at plant stage T1) was examined, as well as a limited set of 40 genotypes at the plant stages of 10 days (EC 12; T1), 5 weeks (EC 19; T2), and 10 weeks (EC 25; T3). Distribution of leaf rust resistance for different sets are given by mean value and range of infected leaf area.

	Analysis with Whole Data Set	All Genotypes of Plant Stage T1	T1	T2	T3
No. of genotypes	240	240	40	40	41
No. inoculation groups	18	12	12	2	2
Range of infected leaf area (%)	0.3–26.7	0.6–2.6	0.6–2.4	0.3–26.7	0.5–5.5
Mean of infected leaf area (%)	1.4	1.1	1.1	3.6	1.0
$\sigma_{Genotype}^{2}$	22.2%	11.9%	13.7%	82.8%	31.0%
$\sigma_{Genotype\;x\;Age}^{2}$	23.8%	-	-	-	-
$\sigma_{Inoculation\;group}^{2}$	12.9%	21.5%	28.0%	3.0%	8.9%
$\sigma_{Inoculation\;group\;x\;Genotype}^{2}$	14.2%	46.5%	39.9%	0.0%	14.4%
$\sigma_{error}^{2}$	26.9%	20.2%	18.4%	14.2%	45.7%
$h^{2}$	0.64	0.54	0.60	0.92	0.58

**Table 5:** Pearson correlation between seedling and adult plant resistance against leaf rust based on greenhouse and field testing, respectively. The whole genotypic set was tested as 10-day old seedlings (EC 12), while selected genotypes were examined at plant developmental stages T1 (EC 12), T2 (EC 19), and T3 (EC 25) within replicated greenhouse trials.

	All Genotypes at Plant Stage T1	T1	T2	T3
Pearson correlation to field data	0.40 ***	0.48 **	0.39 *	0.34 *
*p*-value	2.6 × 10^−10^	0.0018	0.013	0.032
No. of genotypes	232	40	40	41

Significant at * *p*-value ≤ 0.05, ** ≤ 0.01, *** ≤ 0.001. Otherwise, not significant.

**Table 6:** Pearson correlation of leaf rust scores between a single environment and the corresponding serial mean except this examined environment. The number of overlapping genotypes is given in brackets.

	Correlation To Series 2017	Correlation To Series 2018
Nasenberg	0.63 *** (128)	0.63 *** (128)
Nörvenich	-	0.13 (128)
Seligenstadt	0.69 *** (64)	-
Seligenstadt1	0.64 *** (780)	0.74 *** (1008)
Söhnke-Nissenkoog	0.23 (56)	-
Wetze	0.58 *** (104)	-
Wetze1	0.62 *** (780)	0.62 *** (64)
Wetze2	-	0.72 *** (1008)
Wohlde	0.58 *** (352)	-

Significant at *** *p*-value ≤ 0.001. Otherwise, not significant.

## Data Availability

The datasets generated for this study are available in the Supplementary Materials.

## Supplementary Materials

The following are available online at https://www.mdpi.com/article/10.3390/biology10070628/s1, Figure S1: Example images of deatched leaf assays to visualize the wide range of leaf rust infestation within greenhouse experiments. Each column includes the same genotype, while the given percentage of infected leaf area (Infected area) was analyzed by the BluVison software. Infestation data of tested wheat lines (B) were confronted with results of the susceptible control *Borenos* (A). For control means <2% infected leaf area per inoculation group, the whole inoculation group was excluded from statistical analyses, Table S1: Results of leaf rust screening of 232 winter wheat lines and susceptible control *Borenos*. Phenotyping in the field was realized at the date of flowering (EC 65) following an ordinal scale of increasing infestation from 1 to 9. The whole genotype set was examined within greenhouse trials as two leaf seedlings (EC 12; T1). In addition, a limited set of 40 genotypes was tested 5 weeks (EC 19; T2), and 10 weeks (EC 25; T3) after sowing. Columns T1, T2, and T3 give the percentage of infected leaf area based on detached leaf assays. Best linear unbiased estimations (BLUEs) were estimated for each plant developmental stage (T1–T3), while the belonging standard errors are also given.
