# Supplementary material for: Efficiency of a Seedling Phenotyping Strategy to Support European Wheat Breeding Focusing on Leaf Rust Resistance
Source: Biology (Basel). 2021 Jul 6;10(7):628. doi: 10.3390/biology10070628 (PMC8301088; doi:10.3390/biology10070628)
Supplement: Supplementary file 1 [file biology-10-00628-s001.zip › biology-1265541-supplementary.pdf]

Supplementary Material

Table S1. Results of leaf rust screening of 232 winter wheat lines and susceptible control *Borenos*. Phenotyping in the field was realized after flowering (EC65) following an ordinal scale of increasing infestation from 1 to 9. The whole genotype set was examined within greenhouse trials as two leaf seedlings (EC 12; T1). In addition, a limited set of 40 genotypes was tested five weeks (EC 19; T2), and ten weeks (EC 25; T3) after sowing. Columns T1, T2, and T3 give the percentage of infected leaf area based on detached leaf assays. Best linear unbiased estimations (BLUEs) were predicted for each plant developmental stage (T1-T3), while the belonging standard errors are also given.

Genotype	Field	T1	Standard Error (Predicting T1)	T2	Standard Error (Predicting T2)	T3	Standard Error (Predicting T3)
Borenos	-	2.54	0.59	12.62	1.10	4.47	1.04
Line 1	-0.09	0.63	0.88	0.31	2.02	0.55	1.33
Line 2	0.24	0.71	0.88	0.31	2.02	0.59	1.33
Line 3	0.24	0.63	0.88	0.31	2.02	0.77	1.33
Line 4	0.27	0.71	0.88	0.37	2.02	0.55	1.33
Line 5	0.52	1.01	0.88	0.31	2.02	0.59	1.33
Line 6	0.58	0.71	0.88	0.31	2.02	0.99	1.51
Line 7	0.58	1.32	0.88	4.52	2.02	1.53	1.33
Line 8	0.58	0.74	0.88	0.31	2.02	0.55	1.33
Line 9	0.58	0.68	0.88	0.31	2.02	0.55	1.33
Line 10	0.91	0.58	0.88	0.31	2.02	0.55	1.33
Line 11	0.91	0.63	0.88	0.31	2.02	0.55	1.33
Line 12	1.02	1.03	0.88	2.87	2.02	1.03	1.33
Line 13	1.02	1.13	0.88	7.38	2.02	0.73	1.33
Line 14	1.02	0.95	0.88	0.31	2.02	0.55	1.33
Line 15	1.52	0.89	0.88	0.31	2.02	0.55	1.33
Line 16	1.52	0.89	0.88	0.44	2.02	0.59	1.33
Line 17	1.52	0.96	0.88	0.45	2.02	0.55	1.33
Line 18	1.52	1.03	0.88	6.92	2.02	1.03	1.33
Line 19	1.58	0.58	0.88	0.37	2.02	0.55	1.33
Line 20	2.17	1.04	0.88	1.62	2.02	0.70	1.33
Line 21	5.40	1.68	0.88	6.49	2.02	0.84	1.33
Line 22	5.40	0.92	0.88	0.77	2.02	0.59	1.33
Line 23	6.24	1.79	0.88	3.73	2.02	1.30	1.33
Line 24	6.58	2.06	0.88	26.67	2.02	5.51	1.33
Line 25	6.58	1.31	0.88	4.65	2.02	1.21	1.33
Line 26	6.58	0.63	0.88	0.31	2.02	0.99	1.33
Line 27	6.58	0.76	0.88	1.23	2.02	0.59	1.33
Line 28	6.58	2.10	0.88	12.12	2.02	1.46	1.33
Line 29	6.58	1.64	0.88	3.40	2.02	1.93	1.33
Line 30	6.63	1.01	0.88	4.45	2.02	0.73	1.33
Line 31	6.80	0.96	0.93	2.48	2.02	0.55	1.33

Genotype	Field	T1	Standard Error (Predicting T1)	T2	Standard Error (Predicting T2)	T3	Standard Error (Predicting T3)
Line 32	6.91	0.63	0.88	0.37	2.02	0.59	1.33
Line 33	6.91	0.63	0.88	0.31	2.02	0.55	1.33
Line 34	6.91	0.73	0.88	0.90	2.02	0.60	1.33
Line 35	6.91	1.79	0.88	3.00	2.02	0.88	1.33
Line 36	6.91	0.74	0.88	0.70	2.02	0.55	1.33
Line 37	6.91	0.58	0.88	0.31	2.02	0.55	1.33
Line 38	7.24	1.93	0.88	18.79	2.02	2.46	1.33
Line 39	7.24	2.43	0.88	22.74	2.02	5.15	1.33
Line 40	7.58	1.82	0.88	4.19	2.02	1.13	1.33
Line 41	5.91	0.79	0.88	-	-	0.54	1.51
Line 42	0.24	0.71	0.88	-	-	-	-
Line 43	0.24	0.63	0.88	-	-	-	-
Line 44	0.24	0.58	0.88	-	-	-	-
Line 45	0.24	0.63	0.88	-	-	-	-
Line 46	0.24	0.58	0.88	-	-	-	-
Line 47	0.24	0.58	0.88	-	-	-	-
Line 48	0.24	0.63	0.88	-	-	-	-
Line 49	0.29	0.93	0.88	-	-	-	-
Line 50	0.52	0.96	0.93	-	-	-	-
Line 51	0.58	0.63	0.88	-	-	-	-
Line 52	0.58	0.61	0.88	-	-	-	-
Line 53	0.58	0.58	0.88	-	-	-	-
Line 54	0.58	0.66	0.88	-	-	-	-
Line 55	0.58	0.66	0.88	-	-	-	-
Line 56	0.58	0.60	0.88	-	-	-	-
Line 57	0.58	0.58	0.88	-	-	-	-
Line 58	0.58	0.58	0.88	-	-	-	-
Line 59	0.58	0.58	0.88	-	-	-	-
Line 60	0.62	0.95	0.88	-	-	-	-
Line 61	0.62	0.88	0.88	-	-	-	-
Line 62	0.62	0.91	0.88	-	-	-	-
Line 63	0.91	0.71	0.88	-	-	-	-
Line 64	0.91	0.58	0.88	-	-	-	-
Line 65	0.91	0.60	0.88	-	-	-	-
Line 66	0.91	0.66	0.88	-	-	-	-
Line 67	0.91	0.64	0.88	-	-	-	-
Line 68	0.91	0.72	0.88	-	-	-	-
Line 69	0.95	0.95	0.88	-	-	-	-
Line 70	0.95	0.95	0.88	-	-	-	-
Line 71	1.02	0.89	0.88	-	-	-	-

Genotype	Field	T1	Standard Error (Predicting T1)	T2	Standard Error (Predicting T2)	T3	Standard Error (Predicting T3)
Line 72	1.02	0.89	0.88	-	-	-	-
Line 73	1.02	0.96	0.88	-	-	-	-
Line 74	1.02	1.00	0.88	-	-	-	-
Line 75	1.02	0.91	0.88	-	-	-	-
Line 76	1.02	0.96	0.88	-	-	-	-
Line 77	1.02	0.96	0.88	-	-	-	-
Line 78	1.02	0.96	0.88	-	-	-	-
Line 79	1.02	0.96	0.88	-	-	-	-
Line 80	1.02	0.91	0.88	-	-	-	-
Line 81	1.02	0.91	0.88	-	-	-	-
Line 82	1.02	0.91	0.88	-	-	-	-
Line 83	1.02	0.88	0.88	-	-	-	-
Line 84	1.24	0.72	0.88	-	-	-	-
Line 85	1.29	0.96	0.88	-	-	-	-
Line 86	1.52	1.01	0.88	-	-	-	-
Line 87	1.52	0.96	0.88	-	-	-	-
Line 88	1.52	0.91	0.88	-	-	-	-
Line 89	1.52	0.95	0.88	-	-	-	-
Line 90	1.52	0.91	0.88	-	-	-	-
Line 91	1.52	0.95	0.88	-	-	-	-
Line 92	1.52	0.95	0.88	-	-	-	-
Line 93	1.52	0.88	0.88	-	-	-	-
Line 94	1.67	0.72	0.88	-	-	-	-
Line 95	1.83	0.80	0.88	-	-	-	-
Line 96	1.83	0.72	0.88	-	-	-	-
Line 97	2.02	1.06	0.93	-	-	-	-
Line 98	2.02	0.95	0.88	-	-	-	-
Line 99	2.02	1.03	0.93	-	-	-	-
Line 100	2.18	0.72	0.88	-	-	-	-
Line 101	2.20	0.92	0.88	-	-	-	-
Line 102	2.29	0.95	0.88	-	-	-	-
Line 103	2.29	0.92	0.88	-	-	-	-
Line 104	2.29	0.95	0.88	-	-	-	-
Line 105	2.52	1.09	0.88	-	-	-	-
Line 106	2.62	0.88	0.88	-	-	-	-
Line 107	2.62	0.95	0.88	-	-	-	-
Line 108	2.62	0.88	0.88	-	-	-	-
Line 109	2.62	0.88	0.88	-	-	-	-
Line 110	2.62	0.93	0.88	-	-	-	-
Line 111	2.77	0.90	0.88	-	-	-	-

Genotype	Field	T1	Standard Error (Predicting T1)	T2	Standard Error (Predicting T2)	T3	Standard Error (Predicting T3)
Line 112	2.95	0.91	0.88	-	-	-	-
Line 113	2.95	1.09	0.88	-	-	-	-
Line 114	2.95	0.92	0.88	-	-	-	-
Line 115	2.95	0.99	0.88	-	-	-	-
Line 116	2.95	1.31	0.93	-	-	-	-
Line 117	3.02	1.01	0.88	-	-	-	-
Line 118	3.02	1.18	0.88	-	-	-	-
Line 119	3.02	0.88	0.88	-	-	-	-
Line 120	3.02	1.16	0.88	-	-	-	-
Line 121	3.02	0.92	0.88	-	-	-	-
Line 122	3.02	0.92	0.88	-	-	-	-
Line 123	3.16	0.92	0.88	-	-	-	-
Line 124	3.16	0.90	0.88	-	-	-	-
Line 125	3.24	0.61	0.88	-	-	-	-
Line 126	3.24	0.71	0.88	-	-	-	-
Line 127	3.24	0.59	0.88	-	-	-	-
Line 128	3.24	0.71	0.88	-	-	-	-
Line 129	3.24	1.53	0.88	-	-	-	-
Line 130	3.24	1.79	0.88	-	-	-	-
Line 131	3.24	2.63	0.88	-	-	-	-
Line 132	3.24	1.87	0.88	-	-	-	-
Line 133	3.28	0.63	0.88	-	-	-	-
Line 134	3.29	0.92	0.88	-	-	-	-
Line 135	3.29	1.29	0.88	-	-	-	-
Line 136	3.29	0.89	0.88	-	-	-	-
Line 137	3.52	1.68	0.88	-	-	-	-
Line 138	3.52	1.65	0.88	-	-	-	-
Line 139	3.52	1.38	0.88	-	-	-	-
Line 140	3.52	1.11	0.88	-	-	-	-
Line 141	3.52	1.50	0.88	-	-	-	-
Line 142	3.52	0.91	0.88	-	-	-	-
Line 143	3.52	0.99	0.88	-	-	-	-
Line 144	3.58	1.78	0.88	-	-	-	-
Line 145	3.58	1.07	0.88	-	-	-	-
Line 146	3.58	1.72	0.88	-	-	-	-
Line 147	3.58	0.63	0.88	-	-	-	-
Line 148	3.58	0.81	0.88	-	-	-	-
Line 149	3.58	2.18	0.88	-	-	-	-
Line 150	3.58	1.50	0.88	-	-	-	-
Line 151	3.58	1.50	0.88	-	-	-	-

Genotype	Field	T1	Standard Error (Predicting T1)	T2	Standard Error (Predicting T2)	T3	Standard Error (Predicting T3)
Line 152	3.58	0.73	0.88	-	-	-	-
Line 153	3.58	2.25	0.88	-	-	-	-
Line 154	3.58	0.74	0.88	-	-	-	-
Line 155	3.58	0.62	0.88	-	-	-	-
Line 156	3.58	0.60	0.88	-	-	-	-
Line 157	3.58	0.64	0.88	-	-	-	-
Line 158	3.58	2.25	0.88	-	-	-	-
Line 159	3.58	0.85	0.88	-	-	-	-
Line 160	3.62	1.09	0.88	-	-	-	-
Line 161	3.78	0.71	0.88	-	-	-	-
Line 162	3.91	0.66	0.88	-	-	-	-
Line 163	3.91	2.33	0.88	-	-	-	-
Line 164	3.91	0.58	0.88	-	-	-	-
Line 165	4.02	0.91	0.88	-	-	-	-
Line 166	4.02	0.99	0.88	-	-	-	-
Line 167	4.02	1.08	0.88	-	-	-	-
Line 168	4.02	0.96	0.88	-	-	-	-
Line 169	4.20	1.04	0.88	-	-	-	-
Line 170	4.40	1.50	0.88	-	-	-	-
Line 171	4.40	0.90	0.88	-	-	-	-
Line 172	4.52	1.04	0.88	-	-	-	-
Line 173	4.58	0.64	0.88	-	-	-	-
Line 174	4.62	0.88	0.88	-	-	-	-
Line 175	4.62	1.45	0.88	-	-	-	-
Line 176	4.63	0.98	0.88	-	-	-	-
Line 177	4.63	0.96	0.88	-	-	-	-
Line 178	4.67	0.73	0.88	-	-	-	-
Line 179	4.70	0.90	0.88	-	-	-	-
Line 180	4.80	0.96	0.88	-	-	-	-
Line 181	4.83	0.72	0.88	-	-	-	-
Line 182	4.83	2.06	0.88	-	-	-	-
Line 183	4.95	1.19	0.88	-	-	-	-
Line 184	4.95	1.35	0.88	-	-	-	-
Line 185	4.95	0.97	0.88	-	-	-	-
Line 186	4.98	1.06	0.88	-	-	-	-
Line 187	5.00	0.92	0.88	-	-	-	-
Line 188	5.00	0.72	0.88	-	-	-	-
Line 189	5.02	1.01	0.88	-	-	-	-
Line 190	5.17	1.32	0.88	-	-	-	-
Line 191	5.20	0.96	0.88	-	-	-	-

Genotype	Field	T1	Standard Error (Predicting T1)	T2	Standard Error (Predicting T2)	T3	Standard Error (Predicting T3)
Line 192	5.29	1.64	0.88	-	-	-	-
Line 193	5.29	1.13	0.88	-	-	-	-
Line 194	5.29	1.56	0.88	-	-	-	-
Line 195	5.29	1.58	0.88	-	-	-	-
Line 196	5.37	0.98	0.88	-	-	-	-
Line 197	5.37	1.15	0.88	-	-	-	-
Line 198	5.41	0.94	0.88	-	-	-	-
Line 199	5.52	1.13	0.93	-	-	-	-
Line 200	5.52	2.16	0.88	-	-	-	-
Line 201	5.54	0.92	0.88	-	-	-	-
Line 202	5.78	1.87	0.88	-	-	-	-
Line 203	5.78	1.96	0.88	-	-	-	-
Line 204	5.87	1.63	0.88	-	-	-	-
Line 205	5.88	0.97	0.88	-	-	-	-
Line 206	5.91	0.63	0.88	-	-	-	-
Line 207	5.91	0.60	0.88	-	-	-	-
Line 208	5.91	2.22	0.88	-	-	-	-
Line 209	5.91	0.60	0.88	-	-	-	-
Line 210	5.91	0.66	0.88	-	-	-	-
Line 211	5.91	2.40	0.88	-	-	-	-
Line 212	5.91	1.26	0.88	-	-	-	-
Line 213	5.91	1.02	0.88	-	-	-	-
Line 214	5.95	1.10	0.88	-	-	-	-
Line 215	6.02	1.23	0.88	-	-	-	-
Line 216	6.24	2.44	0.88	-	-	-	-
Line 217	6.24	2.68	0.88	-	-	-	-
Line 218	6.24	1.48	0.88	-	-	-	-
Line 219	6.24	1.81	0.88	-	-	-	-
Line 220	6.24	0.66	0.88	-	-	-	-
Line 221	6.24	0.66	0.88	-	-	-	-
Line 222	6.24	1.33	0.88	-	-	-	-
Line 223	6.24	0.60	0.88	-	-	-	-
Line 224	6.24	0.86	0.88	-	-	-	-
Line 225	6.24	0.58	0.88	-	-	-	-
Line 226	6.24	0.76	0.88	-	-	-	-
Line 227	6.24	0.72	0.88	-	-	-	-
Line 228	6.24	1.94	0.88	-	-	-	-
Line 229	6.29	1.13	0.88	-	-	-	-
Line 230	6.52	0.89	0.88	-	-	-	-
Line 231	6.52	1.27	0.88	-	-	-	-

Genotype	Field	T1	Standard Error (Predicting T1)	T2	Standard Error (Predicting T2)	T3	Standard Error (Predicting T3)
Line 232	6.80	1.24	0.88	-	-	-	-
Line 233	7.02	0.98	0.88	-	-	-	-

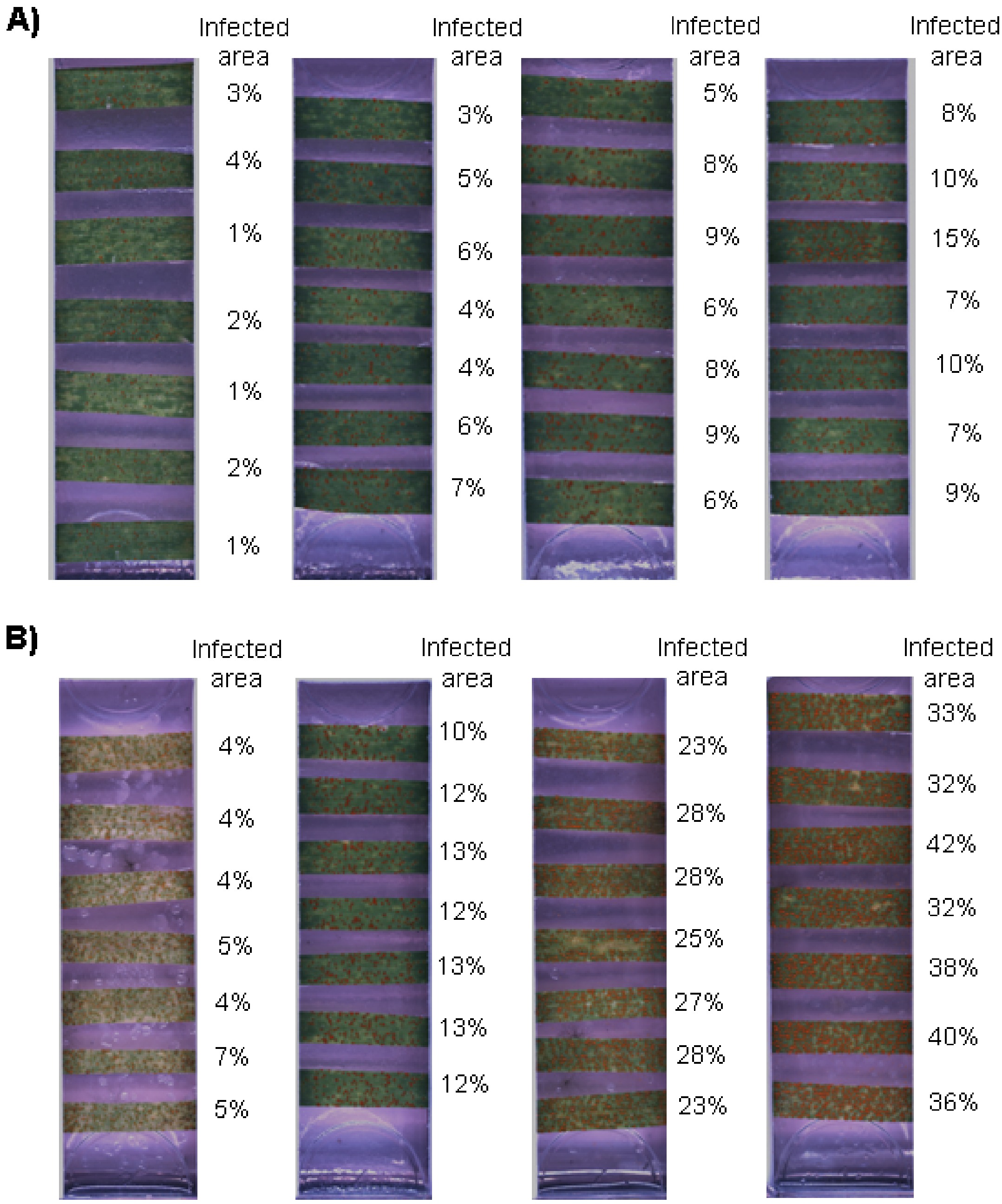

Figure S1: Example images of deatched leaf assays to visualize the wide range of leaf rust infestation within greenhouse experiments. Each column includes the same genotype, while the given percentage of infected leaf area (Infected area) was analyzed by the BluVison software. Infestation data of tested wheat lines (**B**) were confronted with results of the susceptible control *Borenos* (**A**). For control means <2% infected leaf area per inoculation group, the whole inoculation group was excluded from statistical analyses.
